# Multifaceted Role of Extracellular Vesicles: Intercellular Messengers to Therapeutic Applications: A Narrative Review

**DOI:** 10.1002/hsr2.72638

**Published:** 2026-06-18

**Authors:** Kajal Kamra, Satya Kumar Lalam, Shefali Srivastava, Madan Kumar Arumugam, Lokesh Kumar Boopathy, Simran Takkar, Zhiqiu Xia, Sabarinath Peruvemba Subramanian

**Affiliations:** ^1^ Department of Pharmaceutical Sciences University of Nebraska Medical Center Omaha Nebraska USA; ^2^ Department of Pathology, Microbiology and Immunology University of Nebraska Medical Center Omaha Nebraska USA; ^3^ Department of Immunology, Pathology and Infectious Disease University of Nebraska Medical Center Omaha Nebraska USA; ^4^ Cancer Biology Lab, Center for Molecular and Nanomedical Sciences Sathyabama Institute of Science and Technology Chennai Tamil Nadu India; ^5^ Department of Biochemistry JR Medical College and Hospital, BIHER India; ^6^ Department of Biochemistry and Molecular Biology University of Nebraska Medical Center Omaha Nebraska USA; ^7^ Department of Neurological Sciences University of Nebraska Medical Center Omaha Nebraska USA; ^8^ Zoetis Inc. Kalamazoo Michigan USA

**Keywords:** biomarker, disease pathogenesis, extracellular vesicles, intercellular communication, therapeutic applications

## Abstract

**Background and Aims:**

Extracellular vesicles (EVs) are nanoscale carriers, including exosomes, microvesicles, and apoptotic bodies, that mediate intercellular communication by transporting proteins, nucleic acids, lipids, and metabolites. They regulate immunity, tissue repair, and cell differentiation. Increasing evidence highlights their dual roles in disease, where they may promote tumor growth and immune evasion but also serve as diagnostic and therapeutic tools. This review synthesizes advances in EV biology, their roles in health and disease, and their therapeutic potential.

**Methods:**

We conducted a narrative review of recent literature, focusing on EV isolation methods, biological functions, and clinical applications. Sources were selected to capture both mechanistic insights and translational perspectives.

**Results:**

Advances in isolation technologies, from ultracentrifugation to microfluidics, have improved the precision of EV characterization. EVs exhibit distinct molecular signatures, positioning them as promising biomarkers in cancer, cardiovascular, neurodegenerative, autoimmune, and infectious diseases. Mechanistic studies demonstrate their roles in immune modulation, tissue remodeling, and regeneration. As therapeutic agents, EVs show promise as drug delivery systems and as platforms for gene and immunotherapies, offering enhanced targeting and reduced toxicity compared to synthetic nanoparticles. Engineered EVs and hybrid EV–liposome systems further expand their applications, though challenges persist in scalability, standardization, and safety.

**Conclusion:**

EVs are central mediators of intercellular communication with transformative potential in diagnostics and therapeutics. Their biological properties position them as valuable biomarkers and delivery vehicles in precision medicine. Overcoming translational challenges, such as immunogenicity, oncogenic risks, and manufacturing limitations, will be essential for their successful clinical integration.

**Abbreviations**
AF4: asymmetric flow field‐flow fractionation
ARRDC1: arrestin domain‐containing protein 1
BBB: blood–brain barrier
CAF: cancer‐associated fibroblast
CUC: cushioned ultracentrifugation
DEP: dielectrophores
DGC: density gradient ultracentrifugation
DLS: dynamic light scattering
DUC: differential ultracentrifugation
ECM: extracellular matrix
ESCRT: endosomal sorting complex required for transport
EV: extracellular vesicle
FACS: fluorescence‐activated cell sorting
FFF: field‐flow fractionation
HCC: hepatocellular carcinoma
HIF1α: hypoxia‐inducible factor‐1α
I/R: ischemia–reperfusion
MSC: mesenchymal stem cell
MVBs: multivesicular bodies
NK: natural killer
NTA: nanoparticle tracking analysis
PANX1: pannexin 1 channel
ROCK1: Rho‐associated coiled‐coil‐containing protein kinases 1
SEC: size exclusion chromatography
TEM: transmission electron microscopy
TME: tumor microenvironment
Treg: T‐regulatory cell
VEGF: vascular endothelial growth factor

## 1 Introduction to Extracellular Vesicles (EVs)

**Figure 1:**
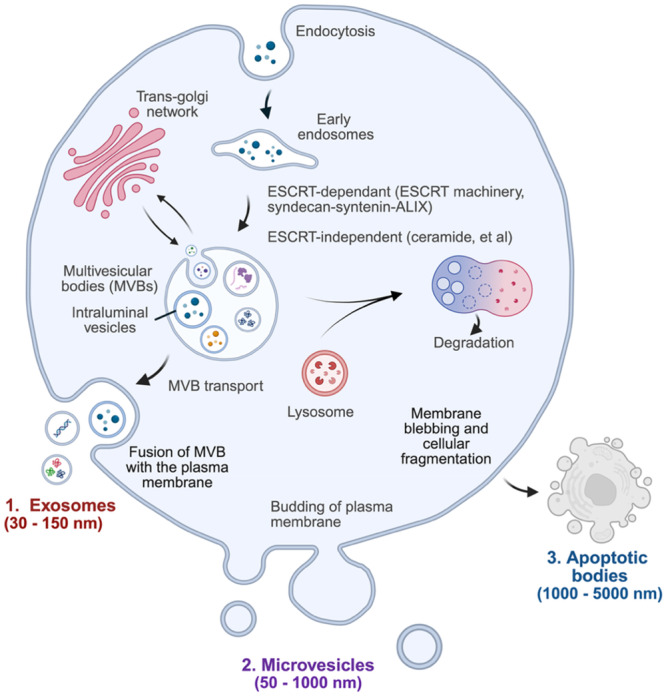
Schematic presentation illustrating the main subtypes of extracellular vesicles (EV). EVs are generated through multiple distinct pathways. (1) Exosome biogenesis: Exosomes form when early endosomes mature into multivesicular bodies (MVBs), which generate intraluminal vesicles (ILVs) through inward budding of the endosomal membrane. MVBs either fuse with the plasma membrane to release ILVs as exosomes or with lysosomes for degradation. (2) Microvesicle biogenesis: Microvesicles are produced by the outward budding and fission of the plasma membrane. (3) Apoptotic body biogenesis: Apoptotic bodies are released during programmed cell death through membrane blebbing.

EVs are lipid bilayer‐bound vesicles measuring between 30 nm and 5 µm (or 5000 nm), released by cells into the extracellular environment. Initially, EVs were considered unwanted cellular debris, and early research primarily focused on their physical attributes like size and morphology [1]. However, EVs are now recognized as vital mediators of intercellular communication involved in various biological processes and disease progression. The biogenesis of EVs includes fundamental intracellular trafficking processes that determine cargo selection and facilitate exocytosis during their release [2]. Different types of EVs have distinct biogenesis mechanisms, resulting in unique characteristics such as size, density, surface proteins, and cargo composition, which are used to classify them into subtypes. The main subtypes include exosomes, microvesicles, and apoptotic bodies (Figure 1).

### 1.1 Types of EVs

Exosomes (30–150 nm) are formed through the inward budding of endosomal compartments, leading to the creation of multivesicular bodies (MVBs) via sequential invagination of the endosomal membrane. When MVBs fuse with the plasma membrane, exosomes are released into the extracellular space [3, 4, 5, 6]. Microvesicles (50–1000 nm), also called ectosomes, are produced by the direct outward budding of the plasma membrane [7]. Apoptotic bodies (1–5 μm or 1000–5000 nm) arise from the fragmentation of cells during programmed cell death [8, 9], however, studies have reported that apoptotic bodies can reach sizes up to 10 μm, particularly those derived from human Jurkat T cells, LIM1215 colon carcinoma cells, and THP‐1 monocytic cells, depending on the characteristics of the parental cells and the biogenesis mechanism [10]. Exosomes are made up of a phospholipid bilayer and contain a variety of cargo, including proteins, nucleic acids (including DNA, mRNAs, miRNAs, and lncRNAs), lipids, and metabolites [11]. Their membrane proteins feature members of the tetraspanin family (CD63, CD9, and CD81), adhesion molecules like integrins, MHC class molecules, and various receptors [12]. The specific cellular origin of different EVs allows them to encapsulate diverse cellular components. Microvesicles primarily carry proteins linked to the plasma membrane and cytosol, reflecting their mode of formation. They also contain heat shock proteins and proteins undergoing post‐translational modifications. In contrast, apoptotic bodies include chromatin, nuclear‐associated proteins, some organelle proteins, and small amounts of glycosylated proteins [12] (Table 1).

**Table 1:** Physiochemical properties of the three main types of extracellular vesicles.

Types	Size	Biogenesis	Markers	Pathways
Exosomes	30–150 nm	Fusion of multivesicular bodies (MVBs) with the plasma membrane	CD9, CD63, CD81, Alix, TSG101, Flotillin, Rab, and ESCRT	ESCRT machinery, syndecan‐syntenin‐ALIX complex, ceramide, tetraspanins, cytoskeletal elements, and arrestin domain‐containing protein 1 (ARRDC1)
Microvesicles	50–1000 nm	Outward budding of plasma membrane	Integrins, selectins, CD40, Annexin A1, CK18, and MMP2	ARF6 and other ARF family members, Rab, Rac1, and RhoA family members, ARRDC1
Apoptotic bodies	1–5 μm (1000–5000 nm) (sometimes up to 10 μm)	Membrane blebbing and cellular fragmentation during apoptosis	Annexin V, phosphatidylserine	Rho‐associated coiled‐coil‐containing protein kinases 1 (ROCK1) and a caspase‐activated pannexin 1 channel (PANX1)

### 1.2 Biogenesis and Sorting of EVs

The biogenesis and sorting of cargo molecules into EVs is regulated by a complex interplay of molecular mechanisms involving various sorting machinery and signaling pathways [5, 13]. Sorting machinery involved in EV biogenesis includes the endosomal sorting complex required for transport (ESCRT), the syndecan‐syntenin‐ALIX complex, tetraspanins, cytoskeletal elements, and arrestin domain‐containing protein 1. The ESCRT machinery is crucial for sorting ubiquitinated proteins into intraluminal vesicles within MVBs, which is essential for exosome formation [13, 14]. Tetraspanins participate in both MVB formation and the packaging of specific cargo into exosomes [15]. The sorting of cargo and shedding of microvesicles are closely linked to the regulation of cytoskeletal elements by several small GTPases, including ARF6, Rab, Rac1, and the RhoA family [16]. Arrestin domain‐containing protein 1 (ARRDC1) also plays a role in the shedding and release of microvesicles from the plasma membrane. Rho‐associated coiled‐coil‐containing protein kinases 1 (ROCK1) and the caspase‐activated pannexin 1 channel (PANX1) have been implicated in the formation of apoptotic bodies [17]. In addition to direct membrane budding during apoptosis, alternative pathways may exist for generating diverse subtypes and releasing apoptotic bodies following fusion with the plasma membrane and MVBs (Table 1). Dysregulation of vesicular sorting mechanisms impacts the cargo composition of specific types of EVs and their functional properties, which can contribute to disease development and progression. Tumor‐derived EVs frequently carry oncogenic proteins and microRNAs, such as EGFRvIII and miR‐1246, which contribute to increased cell proliferation, angiogenesis, and resistance to therapy [18, 19]. In neurodegenerative disorders, such as Alzheimer's disease and Parkinson's disease, EVs mediate the intercellular transfer of misfolded proteins such as tau and α‐synuclein, exacerbating neuronal damage and accelerating disease progression [20, 21]. These pathogenic roles of EVs are discovered to be driven by abnormalities in their sorting machinery, involving the ESCRT complex and RNA‐binding proteins, which have emerged as promising targets for therapeutic intervention [22, 23, 24]. Understanding the unique characteristics of each EV type is crucial for studying the biological function and its application in diagnostics and therapeutics. Hence, current methods for isolating and characterizing EVs, their roles in physiological processes and disease development, their therapeutic potential, the challenges faced in clinical applications, and future research directions are presented.

### 1.3 Methods for EV Isolation

EVs share biophysical properties with supramolecular structures like lipoproteins and protein aggregates. Furthermore, the higher occurrence of these molecules in biological fluids often poses several challenges in the isolation and characterization of EVs. In serum and plasma, lipoproteins, such as HDL and LDL, contaminate EVs because of their similar size and density, affecting the purity of isolated EVs [3]. Tamm–Horsfall protein and albumin bind to EVs and form aggregates that interfere with quantitation [4, 5, 6]. Likewise, casein in milk forms micelles that share a similar size and density with EVs, making the isolation of milk‐derived EVs more intricate [7, 8]. To overcome such challenges, several strategies have been developed to isolate EVs of good purity and maximum yield. Isolation methods leverage distinct physical, biochemical, microfluidic, and immunological properties to separate EVs from extracellular proteins. Several methods exist for isolating EVs, each with distinct tradeoffs in yield, purity, and processing time. Ultracentrifugation, including differential, cushioned, and density gradient variants, separates EVs by density and is widely used but labor‐intensive, taking 4–8 h with low to medium yield. Size exclusion chromatography (SEC) offers a faster, gentler alternative (~20–30 min), with high yield and purity across all biofluids, though it cannot distinguish EV subtypes of similar size. Microfluidics‐based methods, such as dielectrophoresis, affinity isolation, and microfiltration, require minimal sample volumes and can be automated, but may need specialized reagents or frequent maintenance. Field‐flow fractionation (FFF), including asymmetric flow and electric variants, achieves high purity by separating particles using perpendicular force fields, though it demands specialized equipment. Finally, immunocapturing techniques, including microtiter plates, affinity columns, magnetic beads, and fluorescence‐activated cell sorting (FACS), deliver the highest purity by targeting specific surface markers with antibodies but consistently produce very low yields and are best suited for targeted rather than comprehensive EV isolation. Thus, methods for isolating EVs must be suitable, depending on the specific study and purpose. Key distinctions of these methods are summarized in (Table 2).

**Table 2:** Methods of extracellular vesicle Isolation.

Method	Subtypes	Principle	Yield	Purity	Processing times (approx.)	Sample types	Advantages	Limitations	References
Ultracentrifugation	Differential ultracentrifugation (DUC)	Multistep centrifugation based on density typically involves five centrifugation steps to separate EVs from cells, debris, and proteins.	Low	Medium	4–6 h	Plasma, serum, urine, cell culture media	Widely used and considered reliable; Effective for isolating EVs from complex samples.	Labor‐intensive and time‐consuming; Requires large sample volumes; Potential EV damage from high g‐forces; Inconsistent sedimentation due to biological variability and rotor differences.	[25, 26]
	Cushioned ultracentrifugation (CUC)	Utilizes a cushioning medium to protect EVs during centrifugation, enhancing separation based on density differences.	Medium	Medium	5–7 h	Plasma, serum, cell culture media	Better maintains EV integrity compared to DUC; Improved separation from proteins.	Increased workload and cost; Still labor‐intensive.	[27]
	Density gradient ultracentrifugation (DGC)	Employs a density gradient medium (e.g., sucrose, iodixanol) to separate EVs based on their buoyant density during centrifugation.	Medium	Very high	6–8 h	Plasma, serum, CSF, cell culture media	Allows isolation of distinct EV subtypes; Enhanced purity and preservation of EV integrity.	Higher workload and expenses; Requires specialized gradient media; Time‐consuming.	[25]
Size exclusion chromatography (SEC)	qEV columns (Gen 2)	Separates particles based on size using a chromatography column filled with porous beads (e.g., Sepharose, Sephacryl).	High	High	20–30 min	All biofluids (small volumes)	Gentle separation preserving EV integrity; Can be automated and scaled up; Higher purity and yield compared to ultracentrifugation; Quick processing time (approx. 20 min).	Cannot distinguish between EV subtypes of similar sizes; Requires specific resins and columns; Possible co‐elution of similarly sized lipoproteins and protein complexes; Dilution of samples due to mobile phase.	[28, 29]
Microfluidics‐based methods	Dielectrophoresis (DEP)	Manipulates small fluid volumes on a microscale using devices that can separate EVs based on electrical properties.	High	High	10–30 min	All biofluids (small volumes)	Requires minimal sample volumes (as low as 10 µL); Can be automated for high throughput.	Frequent need for de‐clogging filters and channels.	[30]
	Affinity Isolation	Antibody‐based capture on microchannels.	Medium	Very high	1–2 h	Plasma, serum (small volumes)	High recovery rates (ex‐ExoChip achieves > 90% for tetraspanins).	Requires specialized reagents (e.g., antibodies for affinity methods).	[31]
	Microfluidic Filtration	Size‐based filtration through microchannels.	Medium	Medium	15–45 min	Plasma, urine, cell culture media	It's simple, easy to scale, and cost‐effective.	EV loss through adhesion and limited size resolution.	[32]
Field‐flow fractionation (FFF)	Asymmetric flow FFF (AF4)	Separates particles using a perpendicular force field or gradient in combination with a longitudinal flow within a semi‐permeable membrane channel.	High	High	1–2 h	Plasma, serum, conditioned media	Effective separation is based on specific properties like electrophoretic mobility.	Requires specialized equipment and expertise; Not easily implemented with standard lab instruments.	[33]
	Electric FFF	Electric field‐based separation	Medium	High	1‐3 h	Plasma, serum, conditioned media	Can isolate distinct EV populations.	Necessitates sample pre‐concentration; Cannot be used as a standalone method and needs complex setup	[34]
Immunocapturing	Microtiter plate‐based methods	Utilizes antibodies specific to EV surface protein markers to capture and isolate EVs from a mixture(e.g., Enzyme‐linked Immunosorbent Assay).	Very low	High	30–60 min	Plasma, serum (small volumes)	Produces highest purity EV samples.	Lower overall yield compared to size or density‐based methods.	[35]
	Affinity columns	Column‐bound antibodies are used	Very low	Very high	1–2 h	Plasma, serum	Effective for isolating specific EV subpopulations based on surface markers.	Cannot capture all EVs due to reliance on specific markers.	[35]
	Magnetic beads	Antibody‐coated magnetic beads are used	Very low	Very high	1–3 h	Plasma, serum, cell culture media	Produces highest purity for specific EV populations; High recovery efficiency.	Dependent on antibody specificity and availability.	[36]
	Fluorescence‐activated cell sorting (FACS)	Utilizes specific dyes or antibodies that bind to specific molecules on/in the cells, and the cells are sorted based on the emitted fluorescence	Very low	Very high	2–4 h	Plasma, serum (small volumes)	Can sort based on multiple markers simultaneously.	Typically used for targeted studies rather than comprehensive EV isolation; Very low yield and time‐consuming.	[36, 37]

EVs are characterized by examining their size, structure, and molecular content to validate their identity and ensure sample quality [38] (Table 3). To evaluate size and concentration, common methods such as dynamic light scattering (DLS) or nanoparticle tracking analysis (NTA) are used, while transmission electron microscopy (TEM) offers fine‐grained pictures of EV morphology [9]. To confirm EV‐specific proteins, protein markers such as CD9, CD63, and CD81 are identified by mass spectrometry, flow cytometry, or western blotting [11]. Furthermore, EV cargo, including lipids and RNA, is investigated via lipidomics and nucleic acid analysis, which give insight into their functional roles [12]. Characterization guarantees that research and applications based on EVs are reliable.

**Table 3:** Standardized criteria for EV characterization.

Characterization technique	Parameters measured	Sample requirements	Detection range (approx.)	Advantages	Limitations	MISEV2018 recommendation	References
Nanoparticle tracking analysis (NTA)	Size distribution, concentration	10^8^–10^9^ particles/mL	50–1000 nm	Single particle resolution, rapid analysis	Cannot distinguish EVs from other particles	Population analysis	[39, 40, 41]
Transmission electron microscopy (TEM)	Morphology, size, structure	Small volume and fixed samples	1–1000 nm	High resolution, morphological detail	Sample artifacts, time‐consuming	Morphology confirmation	[25, 41, 42]
Western blotting	Specific protein markers	10–50 μg protein	All protein sizes	Quantitative, specific markers	Requires prior knowledge of markers	Marker validation	[41, 43, 44]
Flow cytometry (high‐sensitivity)	Surface markers, heterogeneity	10^6^–10^7^ particles/mL	100–1000 nm	Single vesicle analysis, multiplexing	Size limitations require fluorescent labeling	Single EV analysis	[41, 45, 46]
Dynamic light scattering (DLS)	Hydrodynamic size, polydispersity	High concentration required	1–6000 nm	Rapid, nondestructive	Biased toward larger particles	Size verification	[41, 47]
Resistive pulse sensing (RPS)	Size, concentration	10^6^–10^8^ particles/mL	40–10,000 nm	Single particle counting, tunable pores	Pore clogging, limited throughput	Accurate counting	[29, 41, 48]
Atomic force microscopy (AFM)	Surface topology, mechanical properties	Immobilized samples	1–1000 nm	High resolution, mechanical properties	Sample preparation artifacts	Surface analysis	[41, 49, 50]
Cryo‐electron microscopy	Native structure, morphology	Fresh and unfixed samples	1–1000 nm	Native state preservation	Expensive, specialized expertise required	Native morphology	[41, 51, 52]

### 1.4 Physiological Functions of EVs

EVs in biological fluids play key roles in both physiology and pathology. By initiating signaling cascades, EVs influence gene expression and contribute to physiological functions such as immunomodulation, regeneration, and development [13].

### 1.5 Immunomodulation

**Figure 2:**
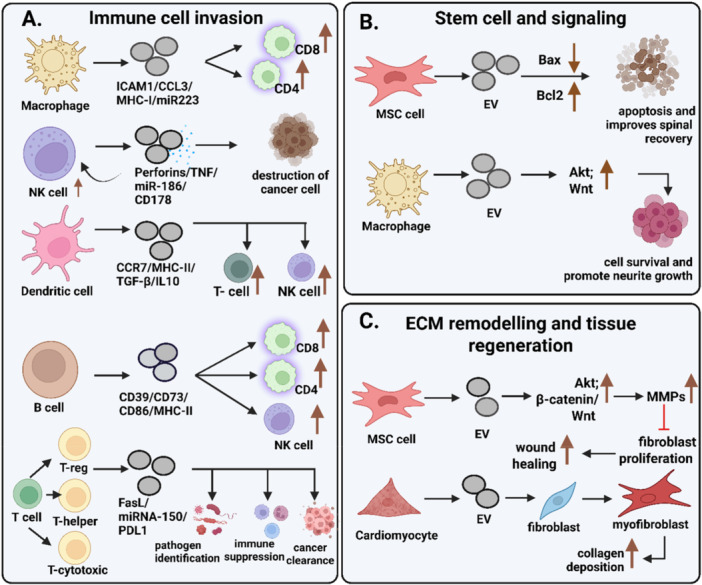
Cellular functions of extracellular vesicles (EVs). (A) Immunomodulatory role of EVs. Immune cell‐derived EVs exhibit diverse properties such as pro‐inflammatory, pro‐apoptotic, cancer surveillance, and immune cell proliferation. (B) Role of EVs in cell signaling. Mesenchymal stem cell‐ and macrophage‐derived EVs have pro‐ and anti‐apoptotic properties by modulating the cell signaling cascade. (C) Role in extracellular matrix remodeling and tissue regeneration. Mesenchymal stem cell‐ and cardiomyocyte‐derived EVs activate Wnt/catenin Akt pathways and differentiation of fibroblasts into myofibroblasts, thus enhancing wound healing and collagen deposition.

Immune cell‐derived EVs modulate the activity of the complement system [14] (Figure 2). While antigen‐presenting cell‐derived EVs inhibit membrane attack complex formation and mononuclear cell‐derived EVs scavenge complement components to prevent complement factors from acting on host cells [15, 16], C‐reactive protein‐bound EVs and T‐cell‐derived EVs activate the C1q pathway [17] and other complement components by phosphorylating complement factor 9 [53]. EVs derived from immune cells play a critical role in modulating the immune responses of neighboring immune cells via cell–cell interactions [54]. Dendritic cell‐derived EVs facilitate antigen presentation, T‐cell activation, and inflammation [55, 56]. Natural killer (NK) cell‐derived EVs exhibit pro‐apoptotic and cytotoxic effects on cancer cells, thus enhancing cancer surveillance and promoting NK cell proliferation [57]. Neutrophil‐derived EVs display both pro‐ and anti‐inflammatory properties, depending on the immune environment [58, 59]. EVs from T cells, including CD4+ helper, CD8^+^ cytotoxic, and T‐regulatory (Treg) cells, play immunomodulatory, cytotoxicity of pathogen‐infected cells, cancer cells and immune‐regulatory functions, respectively. While cytotoxic T‐cell‐derived EVs target non‐self‐peptides, Treg cell‐derived EVs inhibit immune responses [60, 61]. B cell‐derived EVs suppress immune responses by regulating T cells and NK cell activity. Together with T cells, B cell‐derived EVs function as suppressors or promoters of tumor [62]. Macrophage‐derived EVs are influenced by signals from other immune cells. Macrophage‐derived EVs have been shown to play a dual role, as pro‐inflammatory mediators in inflammatory diseases, fibrosis, and cancers, and as mediators of immunoregulation, anti‐tumor, pathogen clearance, and tissue repair [63] (Table 4).

**Table 4:** The mechanistic role of EVs in immunomodulation, tissue remodeling, and regeneration.

Condition	Mechanistic effects	References
Immunomodulation		
Complement system	(A) CD55 accelerates decay of C3 and C5 convertase, preventing complement activation on host cells. (B) CD59 prevents the formation of the membrane attack complex. (C) Complement receptor‐1 (CR1) scavenges complement components from circulation. (D) C‐reactive protein activates C1q of the complement pathway. (E) Casein Kinase 2 Phosphorylates C9, initiating the complement‐mediated pathway.	[16] [64] [65]
Dendritic cells (DCs)	(A) DCs secreted exosomes activate T‐cells by leukocyte function antigen‐1 and ICAM2 signaling pathway. (B) DC‐derived EVs in C57/BL/6 mice induce inflammation in the spleen by the upregulation of the CCR7 chemokine receptor.	[56, 66]
Natural killer (NK) cells	NK cell‐derived exosomes express FasL that regulates the homeostasis of lymphocytes.	[67]
Neutrophils	Neutrophil‐derived EVs contain miR‐451a and miR‐150 that exert anti‐inflammatory roles at the inflammation sites.	[60]
T cells	T‐cell‐derived EVs constitute FasL and APO ligands promoting pro‐apoptotic properties and activating resting CD4^+^ cells.	[68]
T cells	T regulatory cells express miRNA containing exosomes that suppress Th1 cell proliferation and secretion of cytokines.	[62]
B cells	B‐cell‐derived EVs contain high levels of MHC‐II and FasL, which induce apoptosis of helper T cells; B‐cell‐derived EVs contain CD19^+^ and high levels of CD39 and CD73 that inhibit the cytotoxic function of CD8^+^ T cells.	[69]
Macrophage	LncRNAs in macrophages modulate M2 macrophages and lncRNA AFAP1‐AS1 to promote tumor invasion and metastasis.	[70]
Regenerative and tissue repair		
Cell death	In lung injury, monocyte‐derived EVs transport caspase‐1 and casdermin D to vascular endothelial cells of the lungs and stimulate apoptosis; In sepsis, platelet‐derived EVs contain nitric oxide that stimulates caspase‐3 and promotes apoptosis of endothelial cells.	[71] [72]
Cell survival	In spinal cord injury, MSC‐derived EVs enhance functional recovery by upregulating the expression of BCL‐2 and inhibiting the expression of Bax; In myocardial infarction, miRNA‐146a downregulates TNF and IL‐1 receptor‐associated kinase to promote recovery.	[73]
Tissue remodeling		
Fibrosis reversal	Human adipocyte stem‐cell‐derived EVs prevent excessive fibrosis by elevating the ratio of collagen III and TGFβ‐3, which inhibits myofibroblast activity.	[74]
Fibrosis activation	EVs derived from hypoxic tubular cells are enriched in miR‐150‐5p target SOCS1 gene, causing fibroblast activation and upregulating fibronectin expression, which aggravates renal fibrosis.	[75]
Fibrosis activation	Cardiomyocyte‐derived EV contains miR‐208a that promotes the differentiation of fibroblasts into collagen‐secreting myofibroblasts in cardiac tissue.	[76]
Tissue regeneration	Stem cell‐derived EVs stimulate the proliferation and migration of fibroblasts by activating WNT/β‐catenin and Akt signaling pathways.	[77]
Tissue regeneration	Stem cell‐derived EVs stimulate MMPs and phosphorylation of YAP to avoid excessive proliferation of fibroblasts and collagen deposition.	[78]
Tissue regeneration	Hepatocyte‐derived EVs contain ceramide, neutral ceramidase, and sphingosine kinase‐2 to initiate intracellular sphingosine‐1‐phosphate production that promotes hepatocyte proliferation and liver regeneration.	[79]

### 1.6 Regeneration

Tissue regeneration and repair vary across organisms and depend on stem cells' ability to migrate, differentiate, and repopulate damaged tissue. Key mechanisms include apoptosis, inflammation, immune surveillance, matrix remodeling, and cell proliferation [80]. EVs from mesenchymal stem cells (MSCs) play a crucial role by delivering growth factors, miRNAs, and mRNA, enhancing cell survival, regulating apoptosis, and promoting tissue regeneration [81].

EVs exhibit both pro‐ and anti‐apoptotic functions during tissue damage. In acute lung injury, monocyte‐derived EVs deliver caspase‐1 and gasdermin D to lung endothelial cells, promoting apoptosis [82]. In sepsis, platelet‐derived EVs release nitric oxide, activating caspase‐3 and inducing endothelial cell apoptosis [71]. In liver injury, hepatocyte‐derived EVs trigger caspases‐3 and ‐8, along with receptor‐interacting proteins, leading to both apoptosis and necroptosis [72]. In contrast, during acute kidney injury, EVs containing miRNAs (miRNA‐486‐5p and miRNA‐21) promote cell survival by targeting the tumor suppressor PTEN (phosphatase and TENsin homolog deleted on chromosome 10) and enhancing the proliferative response in tubular epithelial cells through CD44‐β–integrin interaction [83, 84]. In spinal cord injury, MSC‐derived EVs improve recovery by increasing anti‐apoptotic proteins (e.g., BCL‐2) and decreasing pro‐apoptotic proteins (e.g., Bax) [85]. Similarly, in myocardial infarction, cardio‐sphere‐derived EVs support cardiomyocyte recovery and prevent tissue necrosis, with miRNA‐146a playing a key role in downregulating inflammation [73]. Neutrophil and macrophage‐derived EVs contain NADPH oxidases, Akt signaling activators, Wnt, and myeloperoxidase, promote neurite growth, intestinal stem cell survival, wound healing, and tissue repair and regeneration [86, 87, 88]. Overall, EVs modulate apoptotic pathways to prevent tissue damage and improve functional outcomes during injuries.

The extracellular matrix (ECM) is composed of collagen, fibronectin, elastin, proteoglycans, and matrix metalloproteinases, providing structural support to tissues. During tissue regeneration, immune cells, fibroblasts, and myofibroblasts actively remodel the ECM. EVs derived from these cells influence ECM remodeling, healing, and scar tissue formation [89]. EVs from human adipocyte stem cells help prevent fibrosis by inhibiting myofibroblast activity and collagen deposition, promoting a favorable collagen‐III to collagen‐I ratio and a TGFβ‐3 to TGFβ‐1 ratio [90]. miRNA‐30a, found in lung spheroid cell‐derived EVs, improves lung fibrosis [74]. In ischemia–reperfusion (I/R) injury, EVs from tubular epithelial cells reduce fibronectin and renal fibrosis, while EVs from hypoxic tubular cells, enriched in miR‐150‐5p, activate fibroblasts, exacerbating fibrosis [91]. Cardiomyocyte‐derived EVs containing miR‐208a promote fibroblast differentiation into collagen‐producing myofibroblasts; blocking miR‐208a reduces fibrosis post‐infarction [75]. Thus, EVs can both support and hinder ECM remodeling during regeneration. Targeting pro‐ and anti‐fibrotic EVs could offer strategies to optimize ECM remodeling, reduce scarring, and enhance tissue regeneration in clinical settings (Table 4).

Stem cell‐derived EVs activate resident cells, promoting their proliferation and differentiation. MSC‐EVs enhance wound healing by stimulating fibroblasts, epidermal cells, and endothelial cells. MSC‐EVs activate the WNT/β‐catenin and Akt pathways to inhibit apoptosis and accelerate healing while regulating scar tissue formation by stimulating matrix metalloproteinases [76] and preventing excessive fibroblast proliferation through YAP phosphorylation [92]. Amniotic fluid stem cell‐derived EVs promote intestinal epithelium regeneration via Wnt signaling [78]. MSC‐EVs and induced pluripotent stem cell‐derived EVs improve cardiac regeneration and function in ischemic heart disease models. Embryonic stem cell‐derived EVs, containing vascular endothelial growth factor (VEGF), activate the PI3K/Akt pathway in endothelial cells, promoting angiogenesis [93]. miRNA‐21 enhances angiogenesis by targeting PTEN, activating Akt/ERK1/2 pathways, and increasing VEGF and HIF‐1α expression [94]. In the brain, MSC‐derived miRNA‐133b supports axonal growth while preventing astrocyte proliferation [95]. In the liver, MSC‐EVs containing lncRNA H19 and miRNA‐17 exhibit anti‐inflammatory and anti‐fibrotic effects, helping reverse fibrosis, stimulate antioxidants, and promote regeneration [96]. Hepatocyte‐derived EVs containing ceramide stimulate sphingosine‐1‐phosphate production, enhancing hepatocyte proliferation and liver regeneration after injury [97]. These examples showcase the tissue repair, proliferation, and regenerative prowess of EVs across multiple organs.

## 2 EVs in Disease Pathogenesis

### 2.1 Cancer

Cancer cells produce EVs, a specialized form of cell communication that contributes to the tumor microenvironment (TME), promotes cancer cell growth and survival, and enhances invasive and metastatic activities [79, 98]. EV secretion is generally higher in malignant cells than in non‐malignant cells, likely due to calcium mobilization from the endoplasmic reticulum and activation of ADP‐ribosylation factor 6 (ARF6) [99]. RhoA [100] regulation of p53 [101], and other oncogenes. Oncogenes such as MYC, RAS, and AURKB have been shown to modulate EV content by enriching them with specific miRNAs (e.g., miR‐21 and miR‐1246), proteins (e.g., integrins and annexins), and long non‐coding RNAs that promote epithelial‐to‐mesenchymal transition (EMT), angiogenesis, and immune evasion [102]. For example, HRASG12V‐transformed cells produce EVs carrying pro‐metastatic miRNAs and altered surface integrins (e.g., integrin αvβ5), which direct EVs to the liver, promoting pre‐metastatic niche formation [102]. Likewise, MYC‐overexpressing cancer cells secrete EVs enriched in miR‐155 and miR‐210, which facilitate angiogenesis and metabolic reprogramming in recipient endothelial and stromal cells [103]. Mechanistically, these oncogenic EVs interact with target cells via receptor‐mediated endocytosis or membrane fusion, delivering their cargo to reprogram gene expression and activate signaling pathways such as PI3K/AKT, MAPK, and TGF‐β, all of which are associated with enhanced invasion and metastasis [102, 104, 105]. Recent studies have emphasized the significant role of EVs in regulating cell signaling across various cancers, including hepatocellular carcinoma (HCC) [106, 107, 108], breast cancer [109], prostate cancer [110, 111], and non‐small cell lung cancer [112]. Hence, EVs play a crucial role in cancer progression by facilitating tumor‐supportive cell signaling, enhancing metastatic potential, and promoting resistance mechanisms against chemotherapy.

### 2.2 Angiogenesis

EVs play an important role in tumor angiogenesis by transporting bioactive molecules such as proteins, lipids, and miRNAs that modulate the TME [113, 114]. Studies have shown that EVs promote endothelial cell proliferation, migration, and tube formation, which are the essential steps in new blood vessel formation [115, 116, 117, 118]. EVs carry angiogenesis‐related miRNAs, such as miR‐21, miR‐155, miR‐210, and miR‐296 [94, 119, 120, 121, 122], lysyl oxidase homolog 4 [123], lncRNA‐H19, and circRNA‐100338 [124, 125], which regulate the expression of pro‐angiogenic factors like VEGF and hypoxia‐inducible factor‐1α (HIF‐1α). Under hypoxic conditions, EVs transfer HIF‐1α to surrounding cells to intensify angiogenesis [126, 127]. Also, tumor‐derived small EVs carry EGF receptors, which are transferred to HUVECs to stimulate VEGF production and blood vessel formation [128, 129, 130]. Furthermore, EVs carry enzymes like matrix metalloproteinases that degrade the ECM, facilitating endothelial cell invasion and sprouting [131]. Hence, understanding the role of EVs in angiogenesis may reveal therapeutic targets to inhibit cancer progression [126].

### 2.3 Neurodegenerative Disorders

EVs play a complex role in the pathogenesis and potential treatment of neurodegenerative disorders [132, 133]. EVs facilitate the spread of misfolded proteins, such as amyloid‐beta, tau, and alpha‐synuclein, contributing to disease progression in Parkinson's disease [134], Alzheimer's disease [135, 136], and other neurodegenerative disorders, including amyotrophic lateral sclerosis [137, 138]. Pro‐inflammatory cytokines, such as IL‐1β and TNF‐α, are carried by EVs generated by activated microglia and astrocytes, aggravating neuroinflammation [139, 140]. Additionally, EVs transport miRNAs and long non‐coding RNAs that regulate gene expression, potentially influencing pathological or protective processes in neurodegenerative diseases [141]. Interestingly, neuroprotective substances, such as brain‐derived neurotrophic factor, can be delivered via EVs derived from stem cells or engineered for therapeutic purposes, showing promise for the regeneration of damaged neural tissues [142, 143].

### 2.4 Cardiovascular Diseases

EVs play a critical role in both physiological and pathological conditions within the cardiovascular system [144]. Cardiovascular EVs facilitate extracellular signaling in both healthy and diseased states, including in vascular pathologies such as atherosclerosis, coronary heart disease, stroke, and peripheral arterial disease [145, 146]. Increasing evidence suggests that the effects of EVs on target cells are primarily dependent on the miRNAs and proteins they transfer, particularly the high expression of miR‐217, miR‐208a, and miR‐27 b‐3p found in pathological conditions [147]. Additionally, EVs from various sources can exert cardioprotective effects in conditions such as myocardial infarction [148], hindlimb ischemia, and stroke by reducing platelet aggregation and inhibiting CD36 expression through ubiquitination, thereby preventing atherosclerotic thrombosis [149, 150].

### 2.5 Infectious Diseases

EVs play a critical role in the pathogenesis and progression of various infectious diseases by mediating intercellular communication and influencing host‐pathogen interactions [151]. EVs can carry viral RNA, proteins, and miRNAs, facilitating immune evasion and viral replication. They also modulate immune responses by affecting T cells and macrophages. Recent studies have shown that EVs are critical to the onset of viral illnesses, including HIV, SARS‐CoV‐2 (COVID‐19), hepatitis B, and hepatitis C [151, 152, 153]. It is well recognized that EVs from eukaryotic cells play a significant role in bacterial infections by influencing both the progression of the infection and the healing timeline [154, 155, 156]. EVs transport fungal antigens and enzymes like aspartyl proteases, which contribute to biofilm formation and immune modulation, leading to severe infections [157].

### 2.6 Autoimmune Disorders

EVs from antigen‐presenting cells such as dendritic cells and macrophages can carry autoantigens, promoting the activation of autoreactive T and B cells, which trigger immune responses in autoimmune disorders like rheumatoid arthritis and systemic lupus erythematosus [158, 159]. EVs with surface antigens can bind to ECM proteins or circulating immunoglobulins, aiding the immune system in recognizing perceived threats, with these responses being mediated by the complement system [160]. Studies have shown that EVs from activated immune cells or damaged tissues carry inflammatory mediators and cytokines, amplifying immune responses in diseases like multiple sclerosis and type 1 diabetes [161, 162, 163, 164].

**Figure 3:**
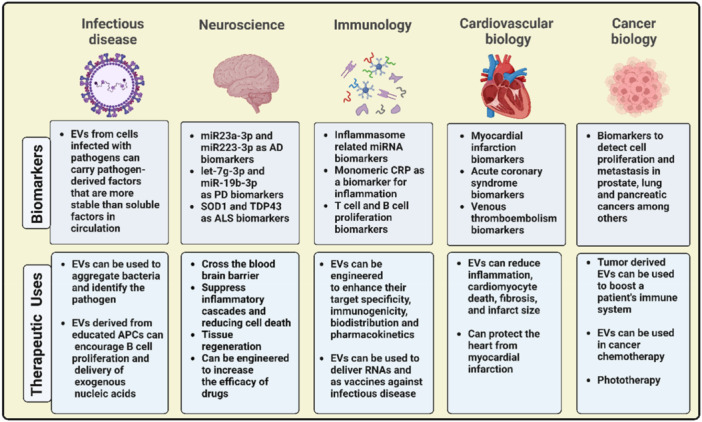
The schematic presentation illustrates extracellular vesicles' therapeutic potential in infectious diseases, neurological disorders, immunology, cardiovascular diseases, and cancer. AD, Alzheimer's disease; ALS, amyotrophic lateral sclerosis; CRP, C‐reactive protein; PD, Parkinson's disease.

In conclusion, EVs are key mediators in diverse pathological processes, including cancer, autoimmune, infectious, and cardiovascular diseases (Figure 3). Deeper insights into EV–disease interactions offer promising avenues for developing diagnostic biomarkers and targeted therapies. Such advances support precision medicine, ultimately improving patient care and outcomes.

## 3 Applications of EVs

### 3.1 Drug Delivery Vehicle

The effectiveness of drug delivery systems largely depends on the characteristics of the delivery vehicle [165, 166, 167]. While liposomes are the best‐established synthetic nanocarrier platform and therefore serve as a common benchmark for comparison, other engineered delivery systems, such as polymeric nanoparticles, dendrimers, and lipid–polymer hybrid nanoparticles, have also been explored for drug delivery applications. In contrast, EVs are gaining increasing attention due to their endogenous origin, inherent biocompatibility, and natural targeting capabilities (Table 5). These differences highlight the unique biological advantages of EVs compared with conventional synthetic nanocarriers. EVs naturally carry proteins, lipids, and nucleic acids and can traverse biological barriers [168]. Molecules like CD47 help them evade immune clearance, while integrins influence organ‐specific targeting [169, 170]. For example, integrin α6β4 and α6β1 promote lung targeting, whereas αvβ5 promotes liver accumulation [169].

**Table 5:** Key differences between extracellular vesicles and liposomes as biological and synthetic nanocarriers for drug delivery.

Feature	Extracellular vesicles	Liposomes
Origin	Naturally secreted vesicles derived from cells	Artificial vesicles composed of synthetic phospholipids
Membrane structure	Phospholipid bilayers contain proteins, lipids, and nucleic acids	The phospholipid bilayer is typically composed of synthetic lipid mixtures
Targeting capability	Intrinsic targeting mediated by membrane proteins (e.g., integrins and tetraspanins)	Targeting requires surface modification (e.g., ligands, antibodies, and PEGylation)
Immune recognition	Generally lower immunogenicity due to endogenous origin	Some formulations may trigger complement activation or rapid clearance by the reticuloendothelial system
Ability to cross biological barriers	Can naturally cross certain biological barriers, such as the blood–brain barrier	Limited intrinsic barrier penetration; often requires ligand modification
Cargo loading	Limited loading capacity: cargo loading methods include electroporation, incubation, and sonication	Higher loading capacity for both hydrophilic and hydrophobic drugs
Composition	Complex composition including proteins, lipids, and nucleic acids from parent cells	Defined synthetic lipid composition
Manufacturing	Isolated from biological sources, scalability remains challenging	Scalable and well‐established manufacturing processes
Clinical maturity	Emerging therapeutic platform	Several clinically approved liposomal formulations

Naturally derived EVs, such as those from MSCs or bovine colostrum, inherently carry bioactive molecules and exhibit therapeutic properties without further modification. In contrast, engineered EVs are modified post‐isolation or via donor cell manipulation to enhance targeting, cargo loading, or therapeutic efficacy. These modifications can include surface functionalization with ligands or incorporation of therapeutic agents, distinguishing them from their natural counterparts [171].

These traits make EVs attractive for delivery, offering longer circulation times, low cytotoxicity, and blood‐brain barrier penetration [171]. Their non‐replicative, nonmutagenic nature offers safety advantages over cell therapies [172]. EVs are being tested as nonviral vectors in gene therapy for neurological, oncologic, and cardiovascular diseases [172, 173, 174].

### 3.2 Therapeutic Applications and Emerging Strategies

EVs have been studied in various gene delivery applications, including pDNAs, siRNAs, mRNAs, antisense oligonucleotides, circRNAs, and CRISPR/Cas9 [175] (Figure 3). Their nanoscale size and flotation density (1.1–1.18 g/mL) favor delivery across barriers like the BBB [176].

Exosomes, a subtype of EVs, show regenerative and immunomodulatory effects. In the nervous system, they support myelin formation, axonal repair, and neuron survival [177, 178]. Exosomes also carry immunologically active cargo. Regulatory T cell‐derived EVs transport anti‐inflammatory miRNAs (miR‐146a‐5p, miR‐150‐5p, and let‐7d) that suppress pro‐inflammatory cytokines [61, 179, 180], modulating immune responses in autoimmunity and cancer.

In cancer, tumor‐derived EVs can propagate metastasis and therapy resistance by transferring oncogenic factors. Hoshino et al. linked tumor‐derived EV integrins to organotropic metastasis [170], stressing the need for rigorous purification to eliminate pro‐tumorigenic contaminants.

In neurodegeneration, EVs can either spread misfolded proteins (tau, α‐synuclein, and Aβ) or deliver neuroprotective cargo from MSCs and neural stem cells [181, 182, 183].

Bioengineered EVs expand therapeutic flexibility. For instance, RVG‐ or GE11‐tagged EVs target brain and EGFR+ tumors via fusion to Lamp2b or CD63 scaffolds [184, 185]. These modifications enhance EV targeting precision and therapeutic efficacy. Similarly, αv‐integrin‐enriched EVs improve lung fibrosis and tumor vasculature targeting [169, 186, 187].

Hybrid EV‐liposome systems combine EV targeting with liposome drug‐loading efficiency. PEG‐mediated fusion enables the co‐delivery of membrane‐bound and soluble drugs while preserving EV membrane proteins [188].

Naturally derived EVs (e.g., from bovine colostrum) are also promising. These vesicles exhibit anti‐inflammatory and antioxidant properties, with good scalability and safety profiles [189, 190].

Several methods are used for therapeutic loading: passive incubation, electroporation, sonication, freeze‐thaw, and extrusion [191]. Electroporation uses electric fields to permeabilize membranes, but it may cause cargo aggregation [192]. Electroporation uses electric fields to permeabilize membranes but may cause cargo aggregation. Sonication aids loading but risks vesicle damage [192]. Optimization is critical to balance efficiency and EV integrity.

Clinical translation requires scalable, reproducible loading methods, coupled with quality control, to manage batch variation and confirm biological activity. These aspects are crucial for clinical trials (Table 6).

**Table 6:** Extracellular vesicle‐based therapeutics in clinical trial.

Condition	Number of subjects	Age (years)	Phase	Recruiting status	NCT number
Ischemic stroke	69	18–75	Phase 1	Not yet recruiting	NCT06612710
Non‐ischemic dilated cardiomyopathies	12	18–80	Phase 1	Recruiting	NCT05774509
Neurodegenerative diseases	100	40–80	Phase1	Not yet recruiting	NCT06607900
Acute respiratory distress syndrome	81	18–85	Phase 1/2	Not yet recruiting	NCT05127122
Diabetic foot ulcers	40	> 18	Phase 2	Recruiting	NCT06319287
Acute myocardial infarction	60	> 18	Phase 4	Completed	NCT02931045
Non‐small cell lung cancer	25	> 19	Phase 2	Completed	NCT03228277
Lung adenocarcinoma	40	> 19	Phase 2	Recruiting	NCT05469022
Chronic post‐COVID‐19 syndrome	60	18–85	Phase 1/2	Not yet recruiting	NCT05116761
Covid‐19 acute respiratory distress syndrome	15	> 18	Phase 1/2	Not yet recruiting	NCT06002841
Bronchopulmonary dysplasia	265	Birth–10 days	Phase 1/2	Recruiting	NCT06279741
Acute respiratory distress syndrome	970	18–75	Phase 3	Recruiting	NCT05354141
COVID‐19	240	18–75	Early Phase 1	Recruiting	NCT05787288
Chronic cough after COVID‐19 infection	80	18–80	Early Phase 1	Recruiting	NCT05808400
Premature ovarian failure	10	20–38 (only females)	Phase 1/2	Recruiting	NCT06202547
Crohn's disease	10	18–75	Phase 1	Active, not recruiting	NCT05130983
Ulcerative colitis	10	18–75	Phase 1	Active, not recruiting	NCT05176366
Retinitis pigmentosa	15	> 18	Phase 1/2	Recruiting	NCT06242379
Perianal fistulizing Crohn's disease	36	18–75	Phase 1/2	Active, not recruiting	NCT05836883
Cochlear implant surgery‐related trauma	11	> 18	Phase 1/2	Not yet recruiting	NCT06545175
Chronic otitis media	100	NA	Phase 2/3	Unknown	NCT04761562
Non‐ischemic dilated cardiomyopathies	12	18–80	Phase 1	Recruiting	NCT05774509
Acute myocardial infarction	60	> 18	Phase 4	Completed	NCT02931045
Neuromyelitis optica spectrum disorders	69	18–65	Phase 1	Not yet recruiting	NCT06620809
Amyotrophic lateral sclerosis	38	18–80	Phase 1/2	Recruiting	NCT06598202

## 4 Challenges and Perspectives

Key translational hurdles remain. Immunogenicity is a concern, especially with repeated or non‐autologous administration [193]. Macrophage‐mediated clearance of EVs has been observed due to recognition of surface proteins and opsonins [194]. Mitigation strategies include expressing CD47 (“don't eat me” signal) or using autologous sources [195]. Immune toxicities like cytokine storms must be preclinically evaluated [196, 197].

Another challenge is the oncogenic risk of TDEVs, which transfer malignant factors and promote metastasis [169, 198]. Quality control and stringent purification are essential to eliminate these risks.

Scalability and standardization are major bottlenecks. Natural EV yields are low, and isolation techniques like ultracentrifugation are laborious. Solutions include high‐density bioreactors, EV‐mimetic nanovesicles, and scalable filtration systems [199], though GMP‐compliant workflows are still evolving.

Formulation and storage stability add complexity. Artificial EVs or EV mimics are being explored to overcome these barriers [188]. Hybrid EV‐liposome fusions offer modularity but require compatible materials. Blocking tumor EV secretion via ESCRT and tetraspanin pathways is also under study [200], though compensatory mechanisms complicate outcomes [201]. EVs have emerged as promising sources of diagnostic and prognostic biomarkers due to their ability to carry disease‐specific nucleic acids, proteins, and lipids that reflect the physiological state of their cells of origin. EV‐associated biomarkers have been identified using a variety of analytical techniques, including quantitative PCR, RNA sequencing, proteomic analysis, and high‐sensitivity flow cytometry. These approaches enable the detection of EV‐derived molecular signatures with potential diagnostic and prognostic value across multiple diseases. Representative examples of EV‐based biomarkers reported across different pathological conditions are summarized in Table 7.

**Table 7:** Extracellular vesicles as biomarkers in pathology.

Condition	EV biomarkers	Detection methods	Experimental models	Phenotypic effects	References
Cancer					
Hepatocellular carcinoma (HCC)	CAFs/miR‐1228‐3p/EVs	EV isolation followed by RT‐qPCR and Western blotting	In vitro HCC9724 cell line; athymic BALB/c nude mouse	This leads to chemoresistance by activating the PI3K/AKT pathway	[202]
HCC	miR‐451/EVs	RT‐qPCR analysis of EV‐derived miRNA	In vitro HepG2, Huh‐7, Hep3B, and SSMC‐772 cell lines	miR‐451a inhibits the proliferation, invasion, and migration, suggesting mIR‐451a is a novel target in HCC	[203]
HCC	CAFs/circZFR/EVs	qRT‐PCR and western blot	In vitro Huh7 and MHCC97L cell lines, athymic nude mouse	circZFR to HCC cells inhibits the STAT3/NF‐KB pathway and promotes HCC development and chemoresistance	[204]
HCC	BMM/miR‐338‐3P	qRT‐PCR and western blot	In vitro Huh7 and MHCC97L cell lines	BMSC‐derived exosome miR‐338‐3p delays HCC by down‐regulating EST1, providing a new promising treatment target for HCC	[205]
Prostate cancer	miR‐424/EVs	RT‐qPCR and miRNA expression analysis	In vitro LNCaP and RWPE‐1 cell lines; Transgenic and PDX mouse	Induces tumorigenesis and stemness in normal epithelial cells	[111]
Lung adenocarcinoma	miR‐23a/EV	RT‐qPCR, TEM, and western blot	In vitro NCI‐H1437, H1648, H1792, and H2087 cell lines; BALB/c nude mouse	Accumulation of HIF1α leads to angiogenesis and vascular permeability, promoting the migration of cancer cells	[206]
Non‐small cell lung cancer	BMSC‐miR‐598/EV	RT‐qPCR	In vitro H1299, A549, H522, and H460 cell lines; BALB/c nude mouse	Decreases migration, invasion, and proliferation of small‐cell lung cancer	[207]
Colorectal cancer	BMSC‐miR‐34a‐5p/EV	RT‐qPCR and western blot	Xenograft BALB/c nude mouse	Decreases tumorigenesis by downregulating myc/PTEN axis	[208]
Pancreatic cancer	BMSC‐EVs/miR‐148a‐3p and engineered EVs (ITGA5‐EVs‐148a)	EV isolation by ultracentrifuge followed by Western blotting and RT‐qPCR detection	In vitro PANC‐1 cell line; BALB/c male nude mice	ITGA5‐EVs‐148a effectively suppressed the proliferation and migration of pancreatic CAFs by targeting ITGA5 through the TGF‐β/SMAD pathway	[209]
Pancreatic cancer	BMSC‐EVs/miR‐338‐5p	Western blotting and RT‐qPCR	In vitro PANC‐1, MIA‐PaCa‐2 and CFPAC1 cell lines; BALB/c nude mouse	Decreases migration, invasion, and tumor formation	[210]
Oral squamous cell carcinoma	CAFs‐ miRNA‐382‐5p/EVs	RT‐qPCR	In vitro CAL‐27 cell line	Induces metastatic potential	[211]
Cardiovascular diseases					
Myocardial infarction	miR‐129‐5p/EVs	RT‐qPCR and western blot	Myocardial infarction induced by coronary artery ligation in C57B/L6	Myocardial infarction mice treated with exosomes overexpressing miR‐129‐5p enhance cardiac function, decrease inflammatory cytokines, and decrease expression of HMGB1.	[212]
Acute myocardial infarction	Islet‐MSC/EV	Western blotting	Acute myocardial infarction induced by coronary artery ligation in C57B/L6	Elevates angiogenesis and survival of endothelial cells	[213]
Ischemic heart disease	Monocyte/MSC‐EV	Western blotting and RT‐qPCR	Myocardial ischemia–reperfusion injury induced by coronary artery ligation in C57B/L6	Elevates the endothelial maturation and cardiac function	[214]
Cardiac ischemia/reperfusion	miRNA‐29c	RT‐qPCR	Ischemia induced in C57B/L6 mice by left anterior descending coronary artery ligation	Decreases autophagy by downregulating PTEN/Akt/mTOR signaling	[215]
Myocardial infarction	BMSC‐miR‐29b‐3bp	RT‐qPCR and TEM analysis	Myocardial infarction induced by left anterior descending coronary artery ligation in SD rat	Increases myocardial angiogenesis	[216]
Atherosclerosis	miR‐23a‐3p/EV	RT‐qPCR	Carotid atherosclerosis is stimulated by a high‐fat, high‐cholesterol diet and partial ligation in SD rat	Accelerate atherogenesis in remote locations by promoting endothelial inflammation	[217]
Fibrosis					
Renal interstitial fibrosis	MicroRNA‐216a/EV	RT‐qPCR	In vitro NRK‐52E cell line; CD‐1 mouse	Activates PTEN/Akt pathway	[218]
Interstitial pulmonary fibrosis	lncRNA/EVs	RT‐qPCR, RNA expression analysis, and western blot	Interstitial pulmonary fibrosis induced by Bleomycin in C57B/L6	Activates lung fibroblasts and induces IPF	[219]
Pulmonary fibrosis	BMSC/EVs	Western blotting, RT‐qPCR, TEM, and NTA analysis	Bleomycin‐induced pulmonary fibrosis model in C57B/L6	Shifts the proinflammatory monocytes to anti‐inflammatory monocytes	[220]
Neurodegenerative diseases					
Alzheimer's disease	MSC‐ microRNA‐29c‐3p/EV	RT‐qPCR and western blot	Alzheimer's disease model induced by injecting oligomer Aβ1–42 in SD rat	Induces Wnt/β‐catenin pathway and ameliorates Alzheimer's disease	[221]
Cerebral ischemia–reperfusion injury	BMSC‐miR‐145	TEM analysis and western blot	BV2 microglial cells and mouse bone marrow MSCs; SD rat	Downregulates FOXO1 expression and infarct area	[222]
Ischemic stroke	BMSC‐EVs	Western blotting, TEM, and NTA analysis	Cerebral ischemia induced by middle cerebral artery occlusion in SD rats	Elevates neurological function and micro‐vessel density	[223]
Brain ischemic injury	BMSC‐EV/miR‐132‐3p	Western blot and NTA analysis	Ischemic stroke induced by transient middle cerebral artery occlusion surgery in C57B/L6	Decreases blood–brain barrier dysfunction and ROS production	[224]

Abbreviations: NTA, nanoparticle tract analysis; TEM, transmission electron microscopy.

With advances in EV engineering, hybrid designs, and biomanufacturing, EV‐based therapeutics may become next‐generation clinical tools if key issues in safety, standardization, and scalability are resolved.

## 5 Conclusion

In conclusion, EVs offer transformative potential in both understanding cellular communication and advancing therapeutic applications. Our paper highlights significant progress in EV research, demonstrating their promise as powerful biomarkers and novel therapeutic tools. Despite challenges related to EV isolation, characterization, and immune responses, ongoing innovations in isolation techniques, standardized characterization, and targeted drug‐delivery methods are paving the way for successful clinical application of EV‐based therapies. While EV‐based strategies offer exciting potential for advancing precision medicine, their clinical impact will depend on overcoming key challenges related to scalability, immunogenicity, and regulatory translation.

## Author Contributions

**Kajal Kamra:** conceptualization, writing – original draft. **Satya Kumar Lalam:** methodology, project administration. **Shefali Srivastava:** investigation, visualization. **Madan Kumar Arumugam:** writing – review and editing, formal analysis. **Lokesh Kumar Boopathy:** conceptualization, writing – review and editing, writing – original draft, supervision. **Simran Takkar:** resources, data curation. **Zhiqiu Xia:** software, methodology. **Sabarinath Peruvemba Subramanian:** investigation, validation.

## Funding

The authors have nothing to report.

## Conflicts of Interest

The authors declare no conflicts of interest.

## Transparency Statement

Dr. Kajal Kamra affirms that this manuscript is an honest, accurate, and transparent account of the study being reported; that no important aspects of the study have been omitted; and that any discrepancies from the study as planned have been explained.

## Data Availability

Data sharing is not applicable to this article as no data sets were generated or analyzed during the current study.
